# Supplementary material for: Development of chemokine network inhibitors using combinatorial saturation mutagenesis
Source: Commun Biol. 2025 Apr 3;8:549. doi: 10.1038/s42003-025-07778-6 (PMC11969024; doi:10.1038/s42003-025-07778-6)
Supplement: Supplementary file 4 — Supplementary Data 2 [file 42003_2025_7778_MOESM4_ESM.zip › SupplementaryData3/scripts/xattune_dr.tar!/./script/analysis/JK/JK_E247_ATHERO_ATC_plot.pdf]

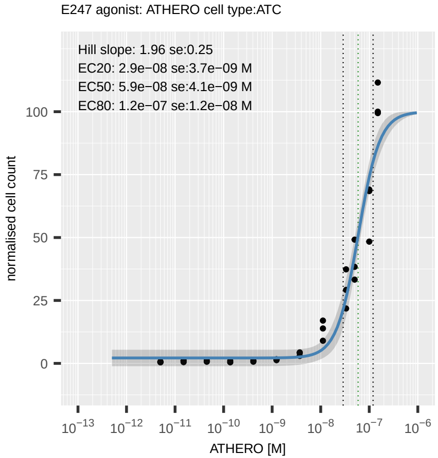

E247 agonist: ATHERO cell type:ATC
Hill slope: 1.96 se:0.25
EC20: 2.9e−08 se:3.7e−09 M
EC50: 5.9e−08 se:4.1e−09 M
EC80: 1.2e−07 se:1.2e−08 M
normalised cell count
100
75
50
25
0
10−13
10−12
10−11
10−10
10−9
10−8
10−7
10−6
ATHERO [M]
